# Farnesol, the farnesol pathway, and the immune-gut-brain axis

**DOI:** 10.3389/fphar.2026.1718322

**Published:** 2026-02-17

**Authors:** Megan Gates, Sean M. Schumacher, William J. Doyle, Natalie Sofaly, Jean-Baptiste Roullet, Javier Ochoa-Repáraz

**Affiliations:** 1 Biomolecular Sciences Graduate Program, Boise State University, Boise, ID, United States; 2 Department of Biological Sciences, Boise State University, Boise, ID, United States; 3 Department of Pharmacotherapy, College of Pharmacy and Pharmaceutical Sciences, Washington State University, Spokane, WA, United States

**Keywords:** farnesol, immune-gut-brain axis, isoprenoids, neuroinflammation, quorum-sensing

## Abstract

Experimental models and clinical evidence suggest that the gut and the central nervous system (CNS) interact in a multifactorial, bidirectional manner. A third player, the immune system, has recently been identified in these interactions, with research linking the gut microbiome to inflammatory conditions, including those affecting the CNS. The molecular signals involved in communication between the gut, brain, and immune system have been extensively studied. However, no unique signaling pathway has been identified for each component of the immune-gut-brain (IGB) axis to date. In this review, we argue that isoprenoids, and specifically farnesol, are key signaling molecules that link the gut and its microbiota, the immune system, and the CNS. The pharmacological properties of farnesol, an intermediate in the broadly conserved mevalonate pathway, are diverse and encompass quorum sensing and microbial biofilm inhibition, neuroinflammatory protection, and modulation of intracellular calcium (Ca^2+^) signaling pathways. Many of these signaling pathways are implicated in neuron-to-neuron communication and in the responses of immunocompetent cells to immunogenic stimuli. We will first address the biological relevance of the immune-gut-brain axis and the gut microbiome in regulating health and disease. Next, we will review the molecular and cellular mechanisms by which farnesol regulates both the gut microbiota and the host’s innate and adaptive immune systems. Finally, we will provide a perspective on the immunoregulatory mechanisms underlying farnesol’s protective properties in models of neuroinflammatory diseases. In summary, we propose a review of the most salient studies that establish farnesol as a significant modulator of the immune-gut-brain axis.

## 1 Introduction

Gut microorganisms, collectively known as the gut microbiota, have been extensively studied in the last 2 decades as key regulators of physiological processes associated with health and disease (Park et al., 2025). Recent advances in molecular biology and bioinformatics, with metabolomics, proteomics, and metagenomics studies, provide extensive taxonomic and functional descriptions of the microbiota (Lin, 2025). Gene profiling and expression studies of the host’s response to microbiota interventions, together with the development of an extensive array of animal models such as germ-free and gnotobiotic rodents, zebrafish, and non-human primates, have complemented omics studies of the microbiota and resulted in significant advances in understanding interactions between the gut microbiota and the host (Nguyen et al., 2015; Li et al., 2025). An increasing number of studies in relevant animal models suggest that gut microbes are involved in neurodevelopment, metabolism, and immune responses. Such studies complement clinical observations of significant differences in gut microbial taxa between healthy and diseased individuals (Durack and Lynch, 2019), suggesting that interactions between microbes and their host are bidirectional (Rathore et al., 2025).

The discovery of crosstalk among the gut microbiota, neurodevelopment, and neuroinflammation has led to the now well-accepted concept of the gut-brain axis, in which neural, metabolic, and immune systems function as its communication pathways. These pathways include the host’s vagus nerve and enteric nervous system, immune and endocrine cells, and neurotransmitters, some of which are co-produced by the host and the microbes (e.g., γ−aminobutyric acid, serotonin). Microbial metabolites are also implicated in host-microbe communications. For example, short-chain fatty acids (SCFAs) have been shown to regulate the integrity of both the intestinal and the blood-brain barriers (Haase et al., 2020; Rahman, 2023). Our team investigates another potential metabolic link between microbes and the host involving the highly conserved mevalonate pathway and one of its downstream metabolites, farnesol. Farnesol was first identified in the flowers of the Farnese acacia tree (*Vachellia farnesiana (L.) Wight & Arn*. [Fabaceae]) (Api et al., 2022). It is also found in essential oils from lemongrass (*Cymbopogon citratus* (DC.) *Stapf* [Poaceae]), citronella (*Cymbopogon nardus (L.) Rendle* [Poaceae]), rose (*Rosa gallica L.* [Rosaceae]), chamomile (*Matricaria recutita* L. [Asteraceae]), among others, also fruits, such as tomato (*Solanum lycopersicum L.* [*syn.: Lycopersicon esculentum Mill.;* Solanaceae]), and in other vegetables (Jung et al., 2018). Farnesol is also produced by yeast and is endogenously synthesized by mammalian cells as part of the mevalonate pathway; it is found in the human brain at concentrations of 110–290 pmol/g (Roullet et al., 1999). Farnesol is pharmacologically active and has demonstrated protective effects on multiple cellular signaling pathways, all of which are involved in the pathogenesis of neuroinflammatory conditions (Leonhardt et al., 2015; De Loof and Schoofs, 2019b; Jo et al., 2021; Park et al., 2021; Sell et al., 2021; Doyle et al., 2023).

The study of the microbiome and of its potential links to disease has led to the widespread use of the concept of dysbiosis, which refers to imbalances in gut microbial composition, function, and the secretion of microbial metabolites. The number of studies listed in PubMed since 2012 on the gut-brain axis exceeds 10,000 (as of the submission of this manuscript), demonstrating a surge in research interest across various fields of neuroscience, including neurodevelopment, neurophysiology, and neuroimmunology. Particularly relevant to this review are studies focusing on the microbiota and the pathogenesis of neuroinflammatory conditions such as multiple sclerosis (MS), and experimental models of the disease, such as experimental autoimmune encephalomyelitis (EAE) (Ochoa-Reparaz et al., 2018; Peters et al., 2024). These models provide a unique opportunity to understand the molecular mechanisms linking microbes to the host brain and its immune system.

The clinical relevance of the gut-brain axis is best exemplified by the recent reclassification of functional gastrointestinal disorders as Disorders of Gut-Brain Interaction (DGBI) in the Rome IV criteria (Schmulson and Drossman, 2017). The reclassification recognizes that conditions, such as irritable bowel syndrome (IBS) and functional dyspepsia, are not only motility disorders but are driven by complex dysfunctions in bidirectional gut-brain communication. Given that DGBI pathophysiology often involves visceral hypersensitivity, mucosal immune activation, dysbiosis, the IGB axis, and potential modulators, this area warrants investigation for these disorders. This review provides an overview of farnesol, its metabolism and derivatives, and its biological relevance as a modulator of the host gut microbiota and immune system. First, we introduce isoprenoids, including farnesol and its derivatives, and discuss their biological activities. Second, we describe the mechanisms by which farnesol may modulate the gut microbiota and discuss its impact on immune responses. Next, we review existing literature on farnesol’s effects on the immune-gut-brain axis, neuroinflammation, and neurodegeneration. We will conclude with a proposed unifying mechanistic hypothesis that the properties of farnesol render the isoprenoid and structural analogues attractive therapeutic options for the treatment of neuroinflammatory conditions and associated dysbiosis.

## 2 Methods

The references for this review were identified through specific searches of PubMed and Google Scholar for articles published from 1995 to date using the terms ‘farnesol’, ‘isoprenoids’, ‘mevalonate pathway’, ‘gut-brain axis’, ‘neuroinflammation’, and ‘biofilm’, among others, depending on the section discussed. Not all articles published with these terms were discussed; the final reference list was generated based on originality and relevance to the broad scope of this review. Priority was given to mechanistic studies that identified specific molecular targets (e.g., calcium channels, nuclear receptors) and to disease models used to assess *in vivo* effects, rather than purely descriptive reports.

## 3 Isoprenoids, farnesol, and farnesol derivatives

### 3.1 Isoprenoids

**FIGURE 1:**
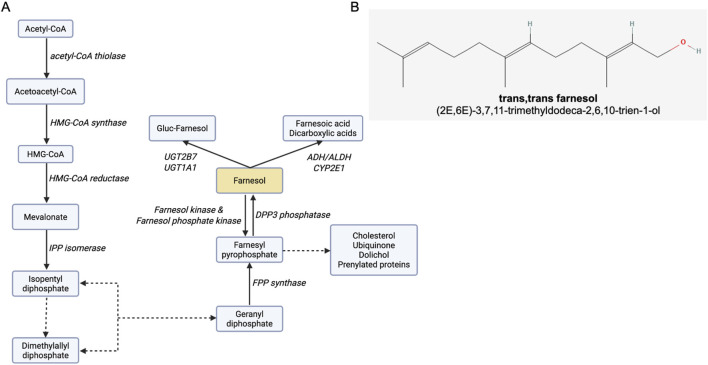
Farnesol and the farnesol pathway. **(A)** The MVA pathway, indicating the enzymes responsible for each metabolic step, and byproducts. Dotted lines represent multistep enzymatic pathways. Abbreviations: ADH, alcohol dehydrogenase; ALDH, aldehyde dehydrogenase; CYP2E1, cytochrome P450 2E1; DPP3, dipeptidyl peptidase 3; FPP, farnesyl pyrophosphate; HMG-CoA, 3-hydroxy-3-methylglutaryl-coenzyme A; IPP, isopentenyl pyrophosphate; UGT1A1, UDP glucuronosyltransferase 1 family, polypeptide A1; UGT2B7, UDP-glucuronosyltransferase-2B7. Adapted from (Roullet et al., 1999 (88). **(B)** Farnesol chemical structure, obtained from: https://pubchem.ncbi.nlm.nih.gov/compound/445070#section=2D-conformer. Image created with Biorender.com.

Isoprenoids are naturally produced organic metabolites found in many prokaryotic and eukaryotic organisms (Clomburg et al., 2019). Approximately 50,000 isoprenoids have been identified, including cholesterol, steroid hormones, carotenoids, vitamin K, farnesol, and farnesol derivatives (Clomburg et al., 2019). They all share a common five-carbon structure that originates from isopentenyl diphosphate (IPP) and dimethylallyl diphosphate (DMAPP). Both IPP and DMAPP are early downstream metabolites of mevalonic acid (MVA) and the methylerythritol 4-phosphate (MEP) pathways (Krause et al., 2020). MVA has been identified in eukaryotes (including yeast and humans), and in some bacteria and archaea, while MEP is found mainly in bacteria and plants (Perez-Gil et al., 2024). The MVA pathway, also referred to as the cholesterol synthesis pathway in mammalian cells, contributes to cell survival. Cholesterol maintains cell membrane integrity, and isoprenoids such as farnesyl-PP and geranylgeranyl-PP ensure intracellular signaling via protein prenylation (Huchelmann et al., 2016). Figure 1A shows the MVA pathway. The MVA pathway has been extensively studied as a drug target, primarily in the context of cardiovascular diseases and blood cholesterol-lowering strategies with statins (Khatiwada and Hong, 2024). The alternative pathway MEP, also known as the non-mevalonate pathway, is found in some bacteria (e.g., *Escherichia coli*), plants, and some eukaryotic microorganisms, such as protozoa and parasites (e.g., *Plasmodium spp*.) (Banerjee and Sharkey, 2014; Guggisberg et al., 2014). MEP also produces IPP and DMAPP, but distinct sets of enzymes regulate these pathways.

The clinical importance of the MVA pathway goes beyond cardiovascular risk reduction. Several other conditions have been linked to the MVA pathway. Some examples include rare genetic disorders such as mevalonate kinase deficiency and Smith-Lemli-Opitz syndrome (Roullet et al., 2012), immune-mediated diseases such as cancers and autoimmunity, and neuroinflammatory and neurodegenerative diseases (Segatto et al., 2019).

### 3.2 Farnesol and farnesol derivatives

Farnesol (3,7,11-trimethyldodeca-2,6,10-trien-1-ol; Figure 1B) is a 15-carbon acyclic sesquiterpene alcohol and a byproduct of the MVA pathway (Faulkner and Jo, 2022), synthesized by the dephosphorylation of farnesyl pyrophosphate (FPP). Several enzymes (e.g., alcohol dehydrogenase, farnesol oxidase) metabolize farnesol to farnesal and farnesoic acid (De Loof and Schoofs, 2019b). Other metabolic pathways have been identified, including glucuronidation by UGT1A1 and UGT2B7 and hydroxylation by CYP2E1 (Singh and Singh, 2025). Reverse pathways converting farnesol back to FPP have been identified and involve several aldo-keto reductases (Endo et al., 2011).

Farnesol structural and metabolic derivatives are biologically active and have been investigated for potential therapeutic applications (Table 1). For example, structural analogues of FPP have been designed to inhibit farnesyltransferase and oncogenic RAS p21 protein prenylation, and investigated as anticancer agents (Dai et al., 2020). Protein prenylation is also implicated in the regulation of inflammation pathways. It has been shown that isoprenoid synthesis and the prenylation of the small G proteins Rac1 and RhoA. RhoA is impaired in mevalonate kinase deficiency, a rare genetic disorder characterized by cyclic episodes of severe pain and inflammation (Politiek and Waterham, 2021; Pisanti et al., 2022). Both small G proteins are key steps in inflammasome activation (Van Der Burgh et al., 2014; Miao et al., 2025). Dysregulated protein prenylation may be implicated in the pathogenesis of additional conditions, including nonalcoholic fatty liver disease (Zhao et al., 2020) and retinopathies (Ashok and Ramachandra Rao, 2024).

**TABLE 1:** Selected farnesol derivatives and biological activities.

Derivative	Biological activities	Refs^a^
Farnesyl pyrophosphate (FPP)	Precursor to farnesol and other key compounds, such as sesquiterpenes, squalene (precursor to cholesterol, which then is precursor to sterols and bile acids), carotenoids, coenzyme Q (essential in the electron transport chain), and dolichol (necessary for protein N-glycosylation, a post-translational protein modification in the endoplasmic reticulum)	Emmerstorfer-Augustin et al. (2016)
	Protein prenylation, as a lipid donor for post-translational farnesylation of proteins involved in membrane anchoring and cell signaling, including small GTPases (Ras and Rho proteins, is essential for cell growth, proliferation, and differentiation	Zhao et al. (2020), Pisanti et al. (2022), Verdaguer et al. (2022)
	Proposed as an agonist for the glucocorticoid receptor, thereby regulating gene expression in inflammation and wound healing	Vukelic et al. (2010)
	Danger signal by leading to calcium influx and acute cell necrosis	Chen et al. (2021)
Farnesyl monophosphate (FMP)	Intermediate to FPP by phosphorylation	Park et al. (2017)
	Intermediate in the “salvage” pathway of isoprenoid biosynthesis (free farnesol is recycled back into the mevalonate pathway)	Hemmerlin (2023)
	Inhibits farnesyltransferase	Clark et al. (2007)
	Modulation of nuclear receptors, such as the peroxisome proliferator-activated receptors (PPARs), is important in lipid metabolism, inflammation, and cellular differentiation. Antagonist of lysophosphatidic acid (LPA) receptors, also important in inflammation	Liliom et al. (2006)
Farnesal (farnesol aldehyde)	Oxidized farnesol by alcohol dehydrogenase serves as an intermediate in insect juvenile hormone, regulating insect development, reproduction, and metamorphosis	Mayoral et al. (2009), Verdaguer et al. (2022)
Farnesoic acid	Oxidized farnesal; it is excreted in animals	Bostedor et al. (1997)
	Proposed cell signaling roles	Falomir-Lockhart et al. (2019)
Farnesyl acetate and other farnesol esters	Inhibits DNA replication *in vitro*	Meigs et al. (1995)
Farnesylated proteins	Cell signaling and Membrane associations	Tamanoi et al. (2001), Basso et al. (2006)
4-Me derivatives of farnesol	Synthetic farnesol methylated analogs	Couillaud et al. (2022)
Trimethylsilyl ether farnesol derivative	Synthetic derivative, used in analytical chemistry for enhanced farnesol detection by gas chromatography	Gupta et al. (2019)
Farnesylamine	Protein farnesylation inhibitor, induces apoptosis, antimicrobial activity and proposed as anticancer therapy)), MAO-B substrate and competitive inhibitor, voltage-gated Ca^2+^ channel inhibitor)	Tanaka et al. (2004), Edmondson et al. (2007), Roullet et al. (2019)

^a^ Refs: References.

Studies have shown that FPP is implicated in neuronal cell death (Chen et al., 2021) by activating nuclear hormone receptors. One of them is the glucocorticoid receptor (Vukelic et al., 2010), a pathway that constitutes another drug target for the treatment of inflammation, cancer, and cocaine addiction. Similarly, farnesyl monophosphate (FMP) modulates peroxisome proliferator-activated receptors (PPARs), directly regulating inflammation and cellular differentiation (Clark et al. (2007)). Studies further suggest that FMP is an antagonist of lysophosphatidic acid (LPA) receptors. LPA receptors are important in regulating cellular migration and proliferation (Wang et al., 2017). Lastly, it is worth noting the therapeutic potential of farnesyl acetate and methyl esters (Table 1) as anticancer agents for their inhibitory activity on cell growth and protein prenylation (Gelb et al., 2006; Verdaguer et al., 2022).

Like farnesol, farnesol derivatives are active on yeast and bacteria. Studies have shown that farnesal and farnesoic acid block the yeast-to-hypha transition and biofilm formation in *Candida albicans* (Nigam et al., 2011; Bell and Chappell, 2014; Gaálová-Radochová et al., 2023). Others have reported that farnesyl acetate, farnesal, farnesoic acid, and farnesyl esters inhibit bacterial growth (Li et al., 2017; Tan et al., 2024b). Overall, current literature suggests that the farnesol pathway is critically involved in microbial biology. This topic will be discussed in more detail in Section 4.

## 4 Farnesol’s molecular targets

Two types of intracellular targets have been identified so far for farnesol. These include nuclear receptors, which are likely responsible for delayed gene-mediated signaling responses to farnesol, and ion channels, which rapidly modulate signaling-dependent responses.

### 4.1 Nuclear receptors

Several studies have demonstrated that farnesol activates nuclear receptors. The first and foundational report on such interaction was by Forman and colleagues (Forman et al., 1995), who identified an orphan nuclear receptor activated by farnesol and its metabolites and named it farnesoid X receptor alpha (FXRα). Activation of the FXR by farnesol regulates lipid and glucose metabolism as well as bile acid synthesis (Baptissart et al., 2013; Chiang and Ferrell, 2022). FXR is primarily expressed in the liver, kidneys, adrenal glands, lungs, and small intestine (Romain et al., 2025). Interestingly, FXR agonists demonstrate anti-inflammatory activity (Grant and DeMorrow, 2020) and improve the clinical presentation of EAE, likely by modulating T- and B-lymphocyte activation and migration (Ho and Steinman, 2016). Further, FXR is expressed in immune cells, including T- and B-lymphocytes, monocytes (Hucke et al., 2016), macrophages (Fu et al., 2022), and dendritic cells (Campbell et al., 2020). FXR is broadly involved in regulating inflammation (Fu et al., 2022; Zhou et al., 2022), intestinal immune responses (Miyazaki et al., 2021; Fu et al., 2022; Zhou et al., 2022), and maintaining the intestinal barrier integrity (Anderson and Gayer, 2021).

Several studies suggest that other nuclear receptors are molecular targets of farnesol, including the thyroid hormone receptor (THR) beta1 and PPARα. Farnesol treatment increased THR expression in a human breast cancer cell line (Duncan and Archer, 2006), and activation of PPARα by farnesol modulates mitochondrial function and energy homeostasis (Cho et al., 2021). PPARα also mediates farnesol’s anti-inflammatory activity through inhibition of the Activator Protein-1 (AP-1) and Nuclear Factor kappa-light-chain-enhancer of activated B cells (NF-κB) transcription factors (Grabacka et al., 2021), suppression of the NLRP3 inflammasome (Alatshan and Benkő, 2021), and upregulation of anti-inflammatory factors, such as β-defensin-1 (Ann et al., 2015).

### 4.2 Farnesol, intracellular calcium signaling, and high-voltage-activated calcium channels

Intracellular calcium concentration is a critical determinant of cell function. Changes in intracellular calcium concentration impact cell growth and differentiation, excitability, exocytosis, and cell death (Bootman and Bultynck, 2020). The endoplasmic reticulum (ER) and mitochondria are two key organelles that contribute to intracellular calcium homeostasis (Panda et al., 2021). Calcium pumps and channels regulate the efflux and influx of Ca^2+^ through the plasma membrane, as well as the movement of Ca^2+^ in and out of the ER and mitochondria (Park and Suh, 2018). The modulation of high-voltage-activated calcium channels (HVA channels) has been thoroughly investigated (Roullet et al., 1999). Farnesol is a pore blocker of HVA channels, inhibiting intracellular calcium signaling. In this section, we examine the effect of farnesol on high-voltage-activated calcium channels. However, this is not to downplay farnesol’s potential to modulate other ion channels, as these are also involved in the homeostasis of intestinal, brain, and immune cells.

Vascular and neuronal HVA channels are inhibited by farnesol, thereby disrupting intracellular calcium signaling (Roullet et al., 1995; Roullet et al., 1996; Roullet et al., 1997b; Roullet et al., 1997a, Roullet et al., 1999). Early studies demonstrate that farnesol blocks L-type channels by binding to and blocking the alpha-1 subunit (Luft et al., 1999). The calcium channel-blocking activity of farnesol is noteworthy, as calcium signaling is a common denominator in microbial and mammalian cell biology. Several studies have shown that VGCC blockers with similar alpha-1-blocking properties (e.g., dihydropyridines) can blunt immune cell activation, microbial growth (e.g., yeast and bacteria), and neuron-to-neuron communication. This suggests that the impact of farnesol on the immune-gut-brain axis may result from a direct inhibition of calcium channels expressed in the cellular components of these systems. This hypothesis will be further developed in the following sections, after the interactions between farnesol, the microbiome, and neuroinflammation have been presented.

Farnesol blocks L-type and N-type voltage-gated calcium channels at micro- (L-type) or nanomolar (N-type) concentrations. Modest inhibition of P/Q and R-type channels has been reported (Roullet et al., 1999). L- and N-type channels are expressed in smooth muscle cells, cardiac cells, and neurons (Zamponi et al., 2015). In neurons, L-type channels control neurite outgrowth and neurotransmission (Morton et al., 2013; Kamijo et al., 2018), whereas N-type channels are found in presynaptic neurons and regulate the secretion of neurotransmitters (Su et al., 2012). L-type channels are also expressed in immune cells, including dendritic cells, macrophages, T lymphocytes, and B lymphocytes (Davenport et al., 2015), suggesting their involvement in both innate and adaptive immune responses. Other VGCC types may also be implicated in immune responses. R-type channels (CaV2.3 channels) are present in dendritic cells and macrophages (Bhandage et al., 2018) as well as in axonal terminals (Dietrich et al., 2003). T-type channels (CaV3s) have been identified in the membranes of CD4^+^ T lymphocytes, where they reportedly regulate granulocyte-macrophage colony-stimulating factor (GM-CSF) secretion, activation of the transcription factor Nuclear Factor of Activated T-cells (NFAT), and autoimmunity (Wang et al., 2016). Notably, CaV3.1 deficiency confers resistance to EAE induction in mice, a protective mechanism attributed to decreased secretion of GM-CSF by pro-inflammatory helper T1 (Th1) and Th17 cells, as well as to inhibition of NFAT signaling (Wang et al., 2016). Whether farnesol inhibits calcium signaling in cells that express L-type calcium channels but are not responsive to changes in membrane potential (e.g., T lymphocytes and dendritic cells) has not been determined. However, like other known calcium channel blockers, the isoprenoid could also inhibit the channels in those cells. Such a possibility represents an attractive testable hypothesis for future investigations. Several other studies indicate that farnesol has a broader role in regulating membrane potential and cell ion balance. Bringman and colleagues reported that farnesol blocks calcium currents mediated by transient low-voltage-activated (LVA) channels, fast Na^+^ channels, and A-type K^+^ channels in retinal glial cells (Bringmann et al., 2000). The blocking of LVA channels by farnesol in other cell types has not been documented. However, since T lymphocytes and microglia have functional LVA channels that regulate cell activation, determining whether farnesol targets these channels in immune cells would be an important step toward understanding the immunomodulatory activity of the isoprenoid (Wang et al., 2016; Tomida et al., 2024). Other ion channels may be targeted by farnesol. Recently, Gc and colleagues demonstrated that farnesol (and geranylgeraniol, a 20-carbon-long isoprenoid) is a positive allosteric agonist of GABA_A_ receptors (GABA_A_Rs) (Gc et al., 2023). GABA_A_R targeting by farnesol is notable because these receptors are activated by microbial GABA and key regulators of gastrointestinal motility and intestinal barrier integrity (Auteri et al., 2015; Belelli et al., 2025).

Calcium channels are not exclusive to mammalian cells. Both prokaryotic and eukaryotic microbes also express them (Verkhratsky and Parpura, 2014; Dong, 2023). The sensitivity (block) of Cch1, a yeast orthologue of the pore-forming α_1_ subunit of mammalian channels, to calcium channel blockers has been documented (Teng et al., 2013). Cch1 shares only 24% of its amino acid sequence with the α_1_ subunit of L-type mammalian channels and lacks a voltage-sensing sequence. However, it forms a high-affinity Ca^2+^-influx complex with Mid1, a N-glycosylated protein that serves as a regulatory subunit of Cch1. Whether farnesol blocks the Cch1-Mid1 complex and calcium signaling in yeast remains unknown, but it is the focus of ongoing research in our laboratory. Studies showing that the yeast-to-hyphae transition is inhibited by verapamil, another clinically approved calcium channel blocker (Yu et al., 2014), suggest it could. The structural similarity between the yeast Cch1 channel and mammalian VGCCs provides a useful molecular framework for investigating isoprenoid-channel interactions. However, while the verapamil-yeast data suggest that farnesol may affect Cch1 channel gating, it is important to note the significant structural differences between the Cch1-Mid1 complex and the multi-subunit architecture of human L-type calcium channels. Experimental confirmation of a direct interaction between farnesol and yeast calcium-selective channels would help distinguish specific channel modulation from broad membrane disruption by farnesol and further our understanding of its role in regulating yeast biology.

The regulation of bacterial calcium signaling by farnesol has not been reported either. Further, the presence of voltage-gated calcium channels in bacteria has received scant attention. To our knowledge, only one recent study reported the presence of functional voltage-gated calcium channels in bacteria, the thermophilic bacterium *Meiothermus ruber* (Shimomura et al., 2020). Interestingly, this channel has a low voltage-activation potential (V_1/2_ of approximately −50 mV), closer to that of mammalian T-type channels, which are maximally open at around −40 mV, and much lower than that of L-type calcium channels, which typically open at −10 mV. Whether farnesol inhibits this bacterial channel remains unknown and warrants further investigation.

## 5 Farnesol as a modulator of microbial biofilms and growth

Studies have demonstrated that farnesol exposure disrupts microbial biofilm formation and inhibits bacterial and fungal growth, potentially contributing significantly to intestinal microbial homeostasis. Improving microbial homeostasis with farnesol may, in turn, regulate inflammatory pathways that are activated in response to dysbiosis, as it has been shown after microbiota interventions, such as antibiotic treatments (Yokote et al., 2008; Ochoa-Repáraz et al., 2009; Seifert et al., 2018), fecal transplantations (Berer et al., 2017; Cekanaviciute et al., 2017; Yoon et al., 2025), and administration of microbiota-derived symbiotic factors (Ochoa-Reparaz et al., 2018) and probiotics (Zangeneh et al., 2025).

Exogenous farnesol exerts significant effects on immune cells, immune responses, and intestinal barrier integrity. It is possible that the immunological effects of farnesol treatment also regulate microbiota composition by altering mucin production in goblet cells or antimicrobial peptide production in Paneth cells, both of which are sensitive to inflammatory mediators (Birchenough et al., 2015; Cui et al., 2023). Another mechanism may be at play here, as studies have shown that host-produced inflammatory cytokines and microRNAs directly affect microbial growth profiles (Liu et al., 2016, Liu et al., 2019; Yuan et al., 2018; Giannoudaki et al., 2019; Narros-Fernández et al., 2024). It is thus possible that farnesol could indirectly improve gut microbial homeostasis by blunting the host’s production of inflammatory cytokines and mRNA.

### 5.1 Farnesol is a biofilm regulator

Biofilms are complex, slime-forming microbial extracellular matrices that create multicellular communities. These communities provide defensive functions against adverse environmental factors, including desiccation, radiation, temperature, and predation. Biofilms are also capable of defense against host innate and adaptive immune factors, including antimicrobial peptides and proteins, or antibodies (Karygianni et al., 2020). The extracellular matrix, known as extrapolymeric matrix substance (EPS), is composed of polysaccharides, proteins, glycoproteins, glycolipids, and extracellular DNA (Karygianni et al., 2020). Biofilms are formed on inert and living substrates through a tightly controlled process involving attachment, the formation of microcolonies, the development of a mature EPS-based biofilm, and ultimately, dissolution and microbial dispersal (Karygianni et al., 2020). Biofilm formation is common surrounding food particles and is attached to the mucus layers of the small and large intestine (Arias and Brito, 2021; Jandl et al., 2024). Chemical and anatomical factors shape microbes’ ability to form biofilms in the gut. These include mucus layer composition (thick in the large intestine), antimicrobial peptide and protein concentrations (high in the small intestine), pH (low in the small intestine), nutrient transit time (slow in the small intestine), and oxygen concentration (low in the large intestine). Accordingly, biofilms are mostly formed in the ileum, in proximity to the colon (Donaldson et al., 2015; McCallum and Tropini, 2024). The microbial composition of the gut biofilms also varies longitudinally (Yang et al., 2025). Disease type and severity also impact intestinal biofilms, as shown in patients with inflammatory bowel disease (IBD) and irritable bowel syndrome (IBS) (Srivastava et al., 2017; Baumgartner et al., 2021).

Quorum-sensing molecules, such as N-acyl homoserine lactones (AHLs) and other autoinducers, are produced by microbes in response to specific environmental stimuli and accumulate extracellularly. At critical concentration, these molecules are recognized by neighboring cells and trigger the expression of specific genes involved in biofilm formation and dissociation. Like other quorum-sensing metabolites, farnesol impacts biofilm formation in bacteria and yeast. In *C. albicans*, farnesol blocks the transition from yeast to hyphae and yeast biofilm formation (Lindsay et al., 2012). The molecular mechanism underlying the effect of farnesol on the yeast-to-hyphae transition is not fully understood but may be mediated by activation of the fungal TEC1 transcription factor (Sachivkina et al., 2020), modification of the yeast morphology secondary to reduced expression of Sap2 and Sap4–Sap6, or changes in the hydrophobicity of the cell membranes that decrease the ability of the yeast to attach to the mucosal surface and initiate biofilm formation (Cao et al., 2005). Farnesol’s quorum-sensing activity has been identified in other *Candida* species (*C. dubliniensis* (Jabra-Rizk et al., 2006), *Candida auris* (Jakab et al., 2021), *C. tropicalis* and *C. krusei* (Bezerra et al., 2020), and *C. glabrata* (Monteiro et al., 2017)), and in other fungal species, such as *Saccharomyces cerevisiae* (Egbe et al., n.d.). However, it has been proposed that increased farnesol production by *C. albicans* is an adaptation that enables it to survive in the highly competitive gut environment (Nickerson et al., 2024). In the gut microbiome, farnesol production by *C. albicans* may modulate its cellular morphology and confer a competitive advantage over other yeasts and bacteria. Farnesol’s effects on *C. albicans* would inhibit yeast-to-hyphal transition, as described above. Although counterintuitive, this would be an adaptation to counter host immune responses, since hyphal stages elicit stronger responses than unicellular forms (Mukaremera et al., 2017). The biofilm inhibition in other yeast species and bacteria, along with its antimicrobial effects, would directly favor *C. albicans*’ competitiveness.

While yeasts are farnesol producers and the MEP pathway for isoprenoid production is also found in most bacteria (Perez-Gil et al., 2024), the extensive literature suggests that most bacteria are not farnesol producers. However, FPP synthase has been identified in *Bacillus subtilis*, and the bacterium may produce farnesol from farnesyl diphosphate (Feng et al., 2014). Nevertheless, although farnesol is not a bacterial quorum-sensing factor, farnesol inhibits bacterial biofilm formation. This has been demonstrated in several bacterial species, including *Staphylococcus aureus* (Jabra-Rizk et al., 2006) and *Streptococcus mutans* (Li et al., 2017). This suggests that intestinal yeast may indirectly regulate intestinal bacterial biofilm formation and gut microbial communities through farnesol production.

### 5.2 Farnesol regulates microbial viability

Although there are many examples of positive interactions between microbes from the same and different domains of life, microbes also employ different mechanisms to outcompete rival species within an environment (e.g., the use of antibiotics). Farnesol is a microbial metabolite that inhibits the growth of other organisms. Farnesol’s antimicrobial activity, along with that of other isoprenoids, has been reported in fungi and bacteria. In *C. albicans*, farnesol reduces the yeast’s ability to invade the host by targeting the unicellular-multicellular transition. However, the molecular mechanisms targeted by farnesol still warrant investigation. Studies in *C. albicans* showed that isoprenoids block the Ras1 protein/cyclic AMP (cAMP)/protein kinase A (PKA) pathway and, in a dose-dependent manner, induce the accumulation of reactive oxygen species (ROS) and apoptosis (Nickerson et al., 2024).

Investigations of the mechanisms underlying farnesol’s antimicrobial activity must critically evaluate their findings, taking into account the concentrations used in their studies. Farnesol’s accumulation in yeast cell membranes has been reported and proposed as a cause of membrane disruption due to its hydrophobicity (Katragkou et al., 2015). It follows that farnesol may have a dual effect on cells: a non-specific toxic activity at high (>100 µM) concentrations and a signaling activity on quorum-sensing pathways at low physiological concentrations (<10 µM). Such functional duality, if confirmed, would have significant implications for therapeutic dosing either with farnesol or with its analogues.

Farnesol acts as an antimicrobial agent against *S. aureus* and potentiates the activity of gentamicin (Jabra-Rizk et al., 2006). The study demonstrated that farnesol activity was dose-dependent: 100 µM significantly enhanced gentamicin efficacy, and higher concentrations (150 µM) were required to induce bacterial death in the absence of gentamicin. The authors concluded that bacterial death resulted from the accumulation of farnesol in bacterial membranes (Jabra-Rizk et al., 2006). Farnesol disruption of the cell membrane integrity results in ion homeostasis imbalances, such as leakage of intracellular potassium ions (K^+^) (Inoue et al., 2004). Furthermore, farnesol exposure results in the death of persisters, non-growing bacterial subpopulations that temporarily resist external insults, such as antibiotics, by acquiring a dormant phenotype (Tan et al., 2024b). Due to the synergistic effects with antibiotics, farnesol has been proposed as a supplement to antibiotic use. There is growing interest in six well-known biofilm-promoting bacteria: *Enterococcus faecium*, *Staphylococcus aureus*, *Klebsiella pneumoniae*, *Acinetobacter baumannii*, *Pseudomonas aeruginosa*, and *Enterobacter spp.,* which are collectively known as the “ESKAPE” group due to their resistance to the most common antibiotics (Tan et al., 2024a, Tan et al., 2025). In 2024, it was reported that farnesol reduced the microbial load and promoted biofilm detachment in the “ESKAPE” biofilm, with no microbes becoming resistant to farnesol even after treatment (Tan et al., 2024a). To date, this is the only mechanistic study investigating the antimicrobial/biofilm properties of farnesols against multiple multidrug-resistant bacteria. In contrast, other groups have focused on individual species within the 6 ESKAPE group (Dolma et al., 2022; Tan et al., 2025). Of note, these studies investigated skin infections and burn wounds and highlighted farnesol’s ability to target bacteria and biofilms. In *Pseudomonas aeruginosa*, farnesol treatment (250 μM) resulted in a reduction of the *Pseudomonas* quinolone signal (PQS) and PQS-controlled virulence factor, pyocyanin (72% decrease) (Cugini et al., 2007). This work also showed reductions in PQS and pyocyanin when *P. aeruginosa* was co-cultured with farnesol-producing *C. albicans*, suggesting the critical role of farnesol in microbial interactions.

## 6 Farnesol impact on immune responses

In this section, we discuss the existing literature on farnesol as a modulator of immune activation, differentiation, and function. We also discuss farnesol’s function as a regulator of cellular survival and proliferation.

### 6.1 Farnesol and the innate immune system

The innate immune system is the body’s rapid, antigen-nonspecific response to external insults, in contrast to the adaptive immune system. The adaptive immune system provides an antigen-specific, more elaborate, evolving response. However, it also delays the response aimed at long-term defense. Activation of the innate system includes the known process of inflammation, which involves responses from tissue barriers, cellular processes, and soluble mediators. Inflammation is an evolutionarily conserved defense mechanism that protects the host’s physiological integrity. However, if uncontrolled or chronically activated, the system becomes a liability to the host, with studies estimating that it is responsible for up to 50% of all deaths worldwide (Furman et al., 2019). The mucosal intestinal tract and other external barriers are constantly exposed to microbes and other insults. The gut mucosal innate immune system operates as a major control center, protecting the physiological integrity of the barrier that separates deeper tissues from the intestinal lumen, its microbes, and microbial products. A metabolic or microbial imbalance can activate this system, leading to local and systemic inflammatory responses, including neuroinflammation (White et al., 2025). The microbiota would then activate the local innate immune system in response to microbial imbalance, propagating inflammation, and amplifying neuroinflammation.

Gut mucosal barriers are essential components of innate immunity, serving as a defense against pathogens and other insults. The gut barrier, a physical and chemical barrier, also serves as the niche for the microbiome, a biological defense mechanism. Studies suggest that farnesol helps maintain the integrity and functionality of the gut mucosal barrier. A recent study demonstrated that farnesol administration reduced dextran sulfate sodium (DSS)-induced colitis in C57BL/6 mice, while maintaining tight junction protein expression levels (Yuan et al., 2024). Another study showed that farnesol promotes upregulation of tight junction proteins (TJP), specifically Zonula Occludens-1 Protein (ZO-1), through activation of the JAK/STAT3 signaling pathway in differentiated, immortalized colorectal Caco-2 cells (Fang et al., 2019). Farnesol could also modulate mucin production, as inflammation regulates its production by goblet cells (Raya Tonetti et al., 2024). The impact of farnesol on the integrity of the intestinal barrier is highly relevant; disruption has been observed in experimental models of neuroinflammatory diseases, including EAE (Nouri et al., 2014; Ahmadi et al., 2020), as well as in humans with neuroinflammatory and neurodegenerative diseases (Camilleri, 2019; Ghezzi et al., 2021; Makdissi et al., 2023).

Pattern recognition receptors (PRRs), such as Toll-like receptors (TLRs), are the primary molecular defense mechanisms identifying external insults. Farnesol has been found to upregulate TLR2 and downregulate TLR4 and TLR6 expression in oral epithelial cells exposed to *Candida albicans* (Décanis et al., 2009). Exposure of epithelial cells to farnesol increased interleukin-6 (IL-6) and β-defensin production (Décanis et al., 2009), suggesting an important immunomodulatory role at the epithelial barrier (Fang et al., 2019). Farnesol’s effects on IL-6 signaling and production were also demonstrated in RAW264.7 cells, used as a surrogate for intestinal macrophages (Ghosh et al., 2010). Dendritic cells (DCs) are also impacted by farnesol. A study showed that farnesol exposure activated immature dendritic cells (iDCs) prior to differentiation, inducing increased CD1d expression (Ghosh et al., 2010). The study also showed that iDCs exposed to farnesol secreted reduced IL-12 and increased IL-10. Farnesol exposure in DCs alters T-lymphocyte differentiation and suppresses adaptive immune responses by downregulating genes critical for mature DC migration (Leonhardt et al., 2015). Interestingly, in the H460 lung carcinoma cell line, farnesol promotes the activation of NF-κB genes, including those encoding inflammatory cytokines such as IL-1, IL-6, and IL-8, as well as cyclooxygenase-2 (COX-2) (Joo et al., 2007). These findings suggest that the homeostatic state of cells conditions their response to farnesol, yielding an inflammatory response in cancerous cells or an anti-inflammatory and immunomodulatory response in resting immune cells.

ROS production is a hallmark of inflammation. ROS generation is a natural process triggered in innate immune cells, particularly phagocytic cells, to facilitate the destruction of engulfed microbes (Sies et al., 2017). ROS are produced by most, if not all, human cell types (Sies et al., 2022). However, their accumulation in cells and tissues secondary to chronic inflammation causes oxidative stress, a phenomenon implicated in the pathogenesis of neuroinflammatory and neurodegenerative diseases (Teleanu et al., 2022; Afzal et al., 2023). ROS themselves trigger inflammation by activating the NF-κB and AP-1 transcription factors (Sallam and Laher, 2016) and upregulating proinflammatory gene expression (Liu et al., 2017). Further, ROS impacts cell growth (Verbon et al., 2012; Patterson et al., 2019), DNA damage response, protein homeostasis, cell survival, and cell death (De Almeida et al., 2022). The wide-ranging impact of ROS on cellular homeostasis has been extensively described in the context of neuroinflammation and neurodegeneration (Teleanu et al., 2022). Interestingly, farnesol can either induce or suppress ROS production. ROS induced by farnesol leads to apoptosis in the fungal pathogens *Aspergillus flavus* (Liu et al., 2010)*,* and *Penicillium expansum* (Fathima Hinaz et al., 2023). As described later in this section, farnesol’s ability to induce ROS production in cancer cell lines is an active topic of research (Fathima Hinaz et al., 2023). However, ROS are also immunomodulatory. In monocyte-derived DCs, ROS production and mitochondrial accumulation via an NADPH-independent mechanism affect sphingolipid metabolism and reduce interferon secretion (Batliner et al., 2024). Such an impact of ROS on interferon synthesis suggests that farnesol-induced ROS production may be the mechanism by which the isoprenoid blunts DC-mediated priming of T cell responses (Leonhardt et al., 2015). However, farnesol-induced *reduction* in ROS levels has also been reported and is associated with *reduced* neuronal death in an acrylamide-induced neurotoxicity rat model (Santhanasabapathy et al., 2015). These seemingly opposing actions of ROS are challenging to interpret, particularly given that the molecular mechanisms by which farnesol regulates ROS production remain largely unknown.

Studies in *Saccharomyces cerevisiae* suggest that farnesol induces mitochondrial membrane depolarization and disrupts the mitochondrial electron transport chain during aerobic respiration (Machida et al., 1998). Other studies in monocyte-derived dendritic cells have implicated interference with sphingolipid metabolism as a mechanism underlying farnesol-induced ROS production and immunomodulation (Batliner et al., 2024). A critical limitation in interpreting the current literature is the wide variance in experimental farnesol concentrations used in published studies. For example, studies reporting activation of farnesol pathways by farnesol have used concentrations equal to or greater than 25 μM. These concentrations are far above those reported in mammalian tissues (Roullet et al., 1999). In contrast, studies demonstrating that farnesol inhibits voltage-gated calcium channels have used low micromolar to sub-micromolar concentrations of the isoprenoid, similar to those reported in tissues. Therapeutic development using farnesol or farnesol derivatives should therefore target these high-affinity interactions to avoid off-target cytotoxicity associated with non-physiological concentrations.

### 6.2 Farnesol and the adaptive immune system

Farnesol also affects adaptive immune responses, both indirectly through its interactions with innate immune cells (antigen-presenting and cytokine-secreting cells) and directly through its modulation of T- and B-lymphocyte function. At low concentration (0.05–5 μM), farnesol decreases the production of IL-2 and IL-5 by murine splenocytes and thus regulates the differentiation of Th1 and Th2 cells (Ku and Lin, 2013, p. 27). These data, along with those discussed in Section 6.1 regarding the impact of farnesol on DCs, suggest an important immunomodulatory role in Th cell differentiation. A direct interaction between farnesol and immunocompetent cells is also possible, considering the demonstrated inhibitory activity of farnesol on L-type calcium-channel mediated intracellular calcium signaling, the presence of such channels in T lymphocytes, and the dependence of T lymphocyte differentiation on intracellular calcium signaling (Badou et al., 2013; Fenninger et al., 2019). To our knowledge, this area of research remains unexplored but offers an exciting opportunity to develop new immunomodulatory treatments for neuroinflammation based on farnesol interactions with L-type calcium channels in immune cells. We discuss next the critical regulatory effects of farnesol on programmed cell death pathways.

### 6.3 Farnesol’s impact on cell survival and apoptosis

Farnesol has been shown to modulate several intracellular pathways implicated in apoptosis and cell death, pathways other than ROS. Exposure of cells to high concentrations of farnesol *in vitro* causes ER stress, mitochondrial dysfunction, and cytochrome c release into the cytoplasm (Joo and Jetten, 2010). These findings have spurred research on the potential use of farnesol as an anti-cancer agent (Rioja et al., 2000; Joo and Jetten, 2010; Joo et al., 2015; Lee et al., 2015). Interestingly, cancer cells are more sensitive to the cytotoxic activity of farnesol than non-cancer cells, such as human T lymphocytes and monocytes isolated from healthy donors (Rioja et al., 2000). The mechanisms underlying this difference between cancer and non-cancer cells are unknown. Still, they may reflect differences in the expression of farnesol-clearing enzymes, leading, in cancer cells, to intracellular accumulation of farnesol to levels that cause cell death. In contrast to the reports in cancer cell lines (Jung et al., 2018), studies in models of neuroinflammation and neurodegeneration suggest that farnesol may have *anti*-apoptotic activity (Santhanasabapathy et al., 2015). Such data on the effect of farnesol on apoptotic pathways are challenging to reconcile. However, known differences in intracellular calcium homeostasis between cancer and non-cancer cells may offer clues to why cancer cells are more sensitive to farnesol. High intracellular calcium concentrations are usually observed in cancer cells and are believed to support uncontrolled growth, survival, migration, and resistance to cell death (apoptosis). Voltage-gated calcium channels (L-, R-, N-, and P/Q-) are expressed in many cancer cell types. Still, their expression is reduced, suggesting that channel inhibition and lowering of intracellular calcium concentrations might be effective therapeutic approaches for treating cancers (Phan et al., 2017). Cancer cell growth dependence on high intracellular calcium levels might make cancer cells more sensitive to farnesol than non-cancer cells. Furthermore, it would make cancer cells more vulnerable to the effects of farnesol on ROS production and on the activation of apoptotic pathways.

The extent to which inflammation influences the expression of voltage-gated calcium channels remains to be fully explored; however, it is well established that calcium channel blockers have anti-inflammatory effects (Hopp et al., 2020; Manzar et al., 2025). This suggests that farnesol-sensitive voltage-gated calcium channels may be present in non-cancer immune cells involved in neuroinflammation and mediate farnesol’s anti-apoptotic activity. Such possibilities and hypotheses warrant further investigation.

## 7 Farnesol regulation of the immune-gut-brain axis

### 7.1 Farnesol’s impact on CNS inflammatory demyelination

The therapeutic potential of farnesol has been explored in models of neuroinflammation. In neuroinflammatory diseases, the immune system mistakenly attacks the CNS, causing inflammation that damages or destroys nerve cells and disrupts communication. MS is a neuroinflammatory disease characterized by demyelination, axonal damage and loss, and neurodegeneration. Remarkably, peptidoglycan, produced only by bacteria, is found in the brains and brain lesions of people with MS (Schrijver, 2001). The presence of peptidoglycan in MS lesions has been proposed as a mechanism underlying neuroinflammation (Laman et al., 2020). Furthermore, specific bacterial strains are found predominantly in the gut of MS patients at early stages of the disease (Rumah et al., 2013). More recently, *Lachnospiraceae* has been identified as a possible key player in the immunopathology of MS and the EAE model of the disease (Yoon et al., 2025). EAE is a CNS inflammatory demyelinating disease that can be studied in non-human primates and rodents (Smith, 2021) and is the most commonly used model to study MS (Constantinescu et al., 2011). Despite the limitations of animal models, EAE shares key pathobiological features with MS. EAE’s hallmarks include local inflammation and immune cell infiltration, as well as axonal demyelination in the brain and spinal cord. While the precise etiology of MS is not fully understood, it is generally accepted that dysregulation of the immune system leads to the breakdown of myelin sheaths in the CNS (Dargahi et al., 2017). EAE was among the first animal models of neuroinflammation evaluated in this context. The role of gut microbes in regulating disease is studied using antibiotics (Yokote et al., 2008; Ochoa-Repáraz et al., 2009; Seifert et al., 2018; Chen et al., 2019a), germ-free mice (Berer et al., 2011; Lee et al., 2011), bacterial reconstitution (Lee et al., 2011), symbiotic factors (Ochoa-Reparaz et al., 2018), bacterial metabolites (Chen et al., 2019b), and probiotics (Lavasani et al., 2010; Mangalam et al., 2017; Calvo-Barreiro et al., 2020; Valizadeh et al., 2021). The gut microbiome has been proposed to confer resistance to EAE in certain rodent strains (Stanisavljević et al., 2018). The microbiome makeup of mice from different sources has been shown to impact disease severity (Daberkow et al., 2021). Furthermore, germ-free EAE mice have been used to study mechanistically how the fecal content of MS individuals modulates EAE severity and immune homeostasis (Berer et al., 2017; Cekanaviciute et al., 2017; Yoon et al., 2025). Probiotics have been proposed as a mechanism of protection against MS (Zangeneh et al., 2025). In addition to rodents, studies in marmoset EAE models have shown that dietary interventions modulate the gut microbiome and disease progression (Kap et al., 2018; Perez-Muñoz et al., 2021). Diet, a primary driver of microbiome composition changes, has been proposed as a mechanism regulating neuroinflammation (Engelenburg et al., 2022; Bronzini et al., 2023).

One proposed target of experimental studies is the imbalance of immune responses triggered in the gut, characterized by increased proinflammatory T helper cells. Increases in Th17 and Th1 responses specifically may drive autoimmune infiltration (Dendrou et al., 2015; Dargahi et al., 2017). Farnesol has shown promising results in controlling EAE severity and inflammatory pathways (Sell et al., 2021; Doyle et al., 2023). EAE mice that were administered daily oral doses of 100 mg/kg farnesol by gavage resulted in a significant reduction in the infiltration of CD4^+^ T cells into the spinal cords compared to untreated mice. MS patients have been found to have reduced frequencies or efficacy of peripheral CD4^+^ regulatory T cells, leaving autoreactive effector T cells unchecked and resulting in an imbalance between Tregs and Th17 cells (Dendrou et al., 2015). Farnesol administration was associated with an increase in Treg frequencies in the spinal cords of EAE-induced mice compared with untreated controls (Sell et al., 2021). Farnesol-treated EAE mice exhibited a significant reduction (∼80%) in disease severity and a delay in the score onset (Sell et al., 2021). Furthermore, our own lab previously performed a transcriptomics analysis comparing gene expression profiles in EAE brains. Results showed downregulation of multiple Th17-cell-associated markers in farnesol-treated mice (oral gavage, 100 mg/kg/day) compared with control mice (Doyle et al., 2023). Additionally, EAE mice treated with farnesol had downregulation of genes associated with oxidative stress, including heat shock response pathways, compared to untreated EAE mice (Doyle et al., 2023). In untreated EAE, ROS are generated, leading to oxidative stress in the CNS and increased T-cell activation. As farnesol attenuates oxidative stress pathways, inflammation is reduced, thereby limiting the adaptive autoimmune response. Furthermore, farnesol treatment significantly reduced the Firmicutes:Bacteroidetes (F:B) ratio by the end of the experiment, and promoted specific alterations in bacterial taxa relative abundances in the gut microbiome compared to sham controls, suggesting that the oral administration of farnesol results in modifications of the gut microbiome in dysbiotic EAE mice (Sell et al., 2021). A lower F:B ratio has been associated with EAE/MS and disease activity, and is considered an indicator of dysbiosis (Chu et al., 2018); however, the biological implications of this association remain to be elucidated. Further studies are required to determine whether farnesol’s effects are direct, via alterations in innate and adaptive immune responses, or indirect, via modification of the gut microbiota, resulting in immunomodulation. As discussed in Section 4, farnesol could effectively disrupt biofilms, virulence mechanisms, and survival of pathogenic microbes. Simultaneously, farnesol could preserve or enrich beneficial gut commensals such as *Lactobacillus* and *Bifidobacterium*. This selective pressure suggests that farnesol could serve as a barrier-protective agent that restores the Firmicutes:Bacteroidetes balance without dysbiosis. Table 2 summarizes the *in vivo* microbiome studies published to date.

**TABLE 2:** Effect of farnesol on gut-relevant bacterial populations and dysbiosis.

Bacterial target/Indicator	Farnesol effects	Model	Proposed mechanisms	Refs^a^
Microbial diversity	Restoration. Reversal of the loss of diversity typically observed in colitis; restoration of community structure	*In Vivo* (DSS-induced Colitis/IBD)	Suppression of colonic inflammation allows recovery of a diverse, homeostatic microbiome	Yuan et al. (2024)
Beneficial commensals (*Lactobacillus* spp., *Bifidobacterium* spp.)	Enrichment. Significant increase in relative abundance of these health-promoting genera	*In Vivo* (Murine EAE model of Multiple Sclerosis)	Modulation of the gut environment to favor anti-inflammatory commensals	Sell et al. (2021)
Dysbiosis Marker (*Firmicutes:Bacteroidetes* Ratio)	Normalization. Reduction of the elevated F/B ratio associated with systemic inflammation	*In Vivo* (Murine EAE & Colitis models)	Selective pressure against Firmicutes expansion; promotion of Bacteroidetes stability	Sell et al. (2021), Yuan et al. (2024)

^a^ Refs: References.

Lack of remyelination is a significant pathological feature of MS. Healthy neurons and oligodendrocytes that undergo acute demyelination are supplied with the necessary cholesterol for repair by astrocytes. In EAE and MS, neuronal cholesterol synthesis is impaired (Berghoff et al., 2021). Transcriptomics in EAE mice confirmed downregulation of cholesterol-synthesis pathways relative to naïve mice (Doyle et al., 2023). In a model of Charcot-Marie-Tooth type 1A, a neurodegenerative inherited disease characterized by myelin loss, administration of farnesol improved rotarod performance, increased motor nerve conduction velocity and compound muscle action potential amplitudes, and enhanced myelination compared with controls (Park et al., 2021). Given farnesol’s ability to affect the MVA pathway (Kaneko et al., 2011), it seems reasonable to believe that farnesol increases cholesterol synthesis in the spinal cord, but further studies are necessary. EAE and demyelination models suggest farnesol’s efficacy against acute autoimmune inflammation; these findings provide a foundation for examining its broader neuroprotective role in chronic, progressive neurodegenerative conditions.

### 7.2 Farnesol’s effects on neurodegenerative diseases

Farnesol exhibits potential multi-faceted neuroprotective properties across experimental models of neurodegeneration, including Parkinson’s and Alzheimer’s diseases. Neurodegenerative disorders, characterized by the progressive loss of neurons leading to physical or mental disability, affect around 15% of the global population (Van Schependom and D’haeseleer, 2023). An increasing number of studies link the immune-gut-brain axis with neurodegeneration (Park and Gao, 2024). Microbial metabolites, such as short-chain fatty acids (SCFAs), tryptophan metabolites, and aromatic amino acids, have been proposed as modulators of blood-brain barrier (BBB) integrity and are suggested to trigger neuroinflammatory processes that precede neurodegeneration (Park and Gao, 2024). As discussed above, bacterial products are found in injured brains and could promote neuroinflammation (Schrijver, 2001; Laman et al., 2020). Because neuroinflammation can lead to neurodegeneration (Adamu et al., 2024), molecules that modulate inflammation, such as farnesol, may confer neuroprotection. Additionally, neuroprotection can also be achieved through direct effects on neurons and glial cells.

The effects of farnesol on neurodegeneration were first evaluated in toxicity models, such as acrylamide-induced (Santhanasabapathy et al., 2015) and lipopolysaccharide-induced neurotoxicity (Santhanasabapathy and Sudhandiran, 2015). In rats subjected to acrylamide-induced neurotoxicity, the oral treatment with farnesol (100 mg/kg/day) compared to controls resulted in reduced ROS levels, astrogliosis, microgliosis, and proinflammatory IL-1β and TNF-α. Farnesol also reduced expression of inducible nitric oxide synthase (iNOS), required for the generation of reactive nitrogen species, in the CNS. Farnesol treatment improved gait, neuromuscular function, and motor coordination in the animals (Santhanasabapathy et al., 2015). In the LPS-induced neurotoxicity model in Swiss albino mice, oral farnesol (100 mg/kg/day) provided experimental neuroprotection via anti-apoptotic mechanisms (Santhanasabapathy and Sudhandiran, 2015). Farnesol administration reduced cytochrome c release from mitochondria, downregulated caspase-3 and Bax, and restored Bcl-2 levels, diminished after subcutaneous LPS administration. Additionally, ROS levels were reduced following farnesol administration in LPS-treated mice (Santhanasabapathy and Sudhandiran, 2015). Farnesol has shown promise as a treatment for neurodegenerative diseases such as Parkinson’s disease (PD) and Alzheimer’s disease (AD). Orally dosed farnesol was found to permeate the BBB in a mouse model of PD, inhibit parkin-acting substrates via protein prenylation, and, in turn, reduce repression of PGC-1α expression (Jo et al., 2021). Further experiments in the same lab also found that muscles weakened by aging or PD neurodegeneration showed increased oxidative metabolism in mice treated with farnesol (Bae et al., 2023). With respect to AD, which is correlated with oxidative stress, excitotoxicity, and mitochondrial dysfunction, the mechanisms of farnesol seem to be a logical match. In a rat model of AD, farnesol treatment showed an overall neuroprotective effect, including recovery of mitochondrial complex activities and reductions in ROS and oxidative damage (Kadian et al., 2024). These findings support the notion that farnesol can enhance antioxidant activity and mitigate neural apoptosis in animal models of neurodegeneration (Santhanasabapathy and Sudhandiran, 2015; Santhanasabapathy et al., 2015). An additional hypothesis regarding the neuroprotective benefit is that farnesol may reduce neuronal apoptosis induced by Ca^2+^ imbalance (De Loof and Schoofs, 2019a). Previous studies have identified the mobilization of Ca^2+^ from the ER to the cytoplasm as a trigger of apoptosis (Viskupicova and Rezbarikova, 2022), which provides a possible mechanistic explanation given farnesol’s previously described role as a voltage-gated calcium channel blocker.

Studies have found that farnesol is also a positive allosteric modulator of γ-aminobutyric acid type A receptors (GABA_A_Rs), altering the binding affinity of GABA to these receptors (Gc et al., 2023). GABA_A_Rs are ligand-gated ion channels located in the postsynaptic membranes of the CNS and are common therapeutic targets for drugs used to treat anxiety, pain, epilepsy, and sedation (Sigel and Steinmann, 2012; Solomon et al., 2019). To our knowledge, investigation into the potential use of farnesol in combination with the drugs has not yet been pursued, but it presents an exciting opportunity. Farnesol has been explored as a potential treatment for epilepsy, given its previously discussed neuroprotective effects (Araújo Delmondes et al., 2022). While farnesol alone did not reduce the overall number of seizures in the mouse model, it increased latency to seizure onset and survival rates. It exhibited antioxidant effects in CNS tissues (Araújo Delmondes et al., 2022).

### 7.3 Farnesol’s effects on neuroinflammation-associated intestinal barrier disruption

Intestinal barrier disruption is a manifestation of neuroinflammatory and neurodegenerative diseases (Ashique et al., 2024). There is no clear answer to the question of whether intestinal barrier disruption precedes neuroinflammation. However, there is strong evidence for bidirectional communication between the gut and the brain, with the immune system serving as a link between them. Impaired intestinal barrier function results from changes in tight junction protein expression, thereby triggering systemic inflammation that amplifies the brain’s inflammatory phenotype (Mu et al., 2017). As discussed in previous sections, Caco-2 cells treated with farnesol showed increased expression of TJPs and enhanced barrier integrity, as measured by transepithelial electrical resistance (Fang et al., 2019). Future research is warranted to investigate whether farnesol directly improves intestinal barrier function in individuals with neuroinflammatory diseases. These effects could reduce gut-mediated systemic inflammation and complement its direct impact on intestinal microbial homeostasis (Sell et al., 2021).

### 7.4 Broader clinical implications

Beyond its role in the immune-gut-brain axis, farnesol shows therapeutic potential for both organic and functional gastrointestinal disorders. In carcinogenesis, dietary farnesol has been shown to inhibit the formation of aberrant crypt foci and reduce crypt multiplicity in rat models of colon cancer (Rao et al., 2002). Regarding functional disorders, especially gastroparesis and functional dyspepsia, farnesol is a key component of Zingiber officinale (ginger), a prokinetic agent (Hu, 2011). Although specific human trials are needed to confirm this, farnesol’s ability to reduce inflammation and its effects on calcium ion channels could provide a plausible mechanistic basis for the effectiveness of farnesol-based therapies in enhancing gastric motility. Additionally, farnesol shows promise in reducing post-surgical complications. Its strong inhibition of Staphylococcal biofilms, as previously discussed, may be relevant to preventing catheter-related bloodstream infections and implant-associated complications. Moreover, recent evidence indicates that farnesol improves intestinal barrier function by upregulating TJPs via the JAK/STAT3 pathway (Fang et al., 2019), suggesting that it may reduce the risk of postoperative bacterial translocation and sepsis.

## 8 Limitations and future perspectives

Farnesol is first and foremost a downstream metabolite of the mevalonate pathway in eukaryotes. In this context, the research suggests that farnesol and the farnesol metabolic pathway are key regulators of the immune-gut-brain axis. However, preclinical murine models have demonstrated therapeutic efficacy. This suggests that farnesol or farnesol analogues may have a role in the therapeutic arsenal for treating disorders of the immune-gut-brain axis. However, translating murine studies to humans presents significant challenges, including issues with bioavailability and metabolic stability.

A critical consideration is farnesol’s pharmacokinetics and potential toxicity. Following oral administration and intestinal absorption, farnesol preferentially distributes to lipid-rich environments and cell membranes, and is metabolized in the liver, kidneys, and intestines (Staines et al., 2004). The metabolic route involves sequential oxidation by alcohol dehydrogenase and farnesol oxidase, producing farnesal and farnesoic acid. Additionally, glucuronidation, mediated by UGT1A1 and UGT2B7, and hydroxylation by CYP2E1 are significant clearance pathways (Staines et al., 2004). Because of its high logP (octanol-water partition coefficient) ranging from 4.16 to 5.31, farnesol is highly hydrophobic. Due to this and its rapid metabolism, farnesol’s bioavailability is low. Regarding its toxicity, *in vivo* studies in rodent models have reported a mean lethal dose (LD_50_) of approximately 5,000 mg/kg of body weight following oral administration (de Araújo Delmondes et al., 2019). Thus, farnesol’s LD_50_ indicates that it is well tolerated at low doses. Higher concentrations may cause irritation upon dermal exposure. However, because of farnesol’s lipophilic nature, vehicles may be required for its administration. Thus, the pharmacokinetics and toxicity of compounds such as organic solvents and oils must also be critically evaluated. Alternative delivery systems and routes of administration of farnesol are being considered. Delivery to the CNS is critical to the management of neurodegenerative diseases. Still, dosing would need to be carefully adjusted to avoid potential cytotoxicity at higher concentrations or complete blockade of neurotransmission. Hence, research would also need to focus on developing targeted delivery systems, such as nano-encapsulation or prodrug formulations, systems that can enhance stability and CNS penetration while mitigating off-target effects. Alternative routes of delivery, such as intra-abdominal administration, could facilitate exposure to specific cellular targets, including APCs. However, delivery systems with low toxicity would be required. Interestingly, encapsulation of farnesol reduces its cytotoxicity (Wu et al., 2019; Wu et al., 2021). Nanoemulsions formulated with farnesol have also been shown to exert immunomodulatory effects on APCs (Mückter et al., 2022). Furthermore, farnesol hydrogels serve as delivery vehicles and have shown promising efficacy in neurodegenerative models, including Parkinson’s disease (Jo et al., 2021), and in tissue repair (Wu et al., 2019).

## 9 Summary

A connection between the digestive system and the brain was proposed back in the mid-1800s (reviewed in (Miller, 2018)). The concept evolved in the 21st century with the inclusion of the gut microbiome as an integral component of the gut-brain connection and the realization that the communication between intestinal microbes and the brain was bidirectional (Margolis et al., 2021; Lewandowska-Pietruszka et al., 2022). However, over the last 2 decades, numerous studies on the gut-brain axis in the context of neuroinflammation and neurodegeneration have highlighted a third contributor to communication between the gut and the brain: the immune system, hence the term immune-gut-brain axis.

**FIGURE 2:**
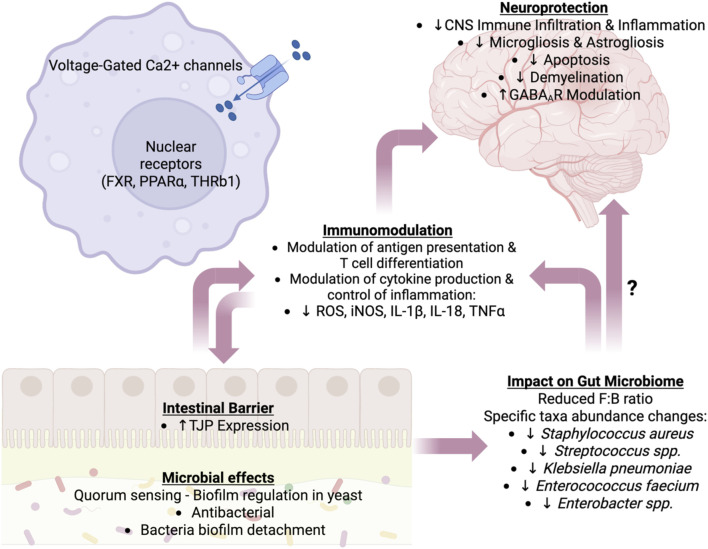
Proposed farnesol’s roles in regulating the immune-gut-brain axis. The effects of farnesol on the host are categorized into neuroprotection, alterations in the gut microbiome, immunomodulation, and modulation of the intestinal barrier. These effects are not unidirectional, and some of farnesol’s impacts on immune, neural, epithelial, and microbial cells are multifactorial, affecting multiple categories. Abbreviations: Ca^2+^, calcium ion; CNS, central nervous system; FXR, farnesoic X receptor; IL, interleukin; GABA_A_R, gamma aminobutyric acid A receptor; iNOS, inducible nitric oxide synthase; PPARα, peroxisome proliferator-activated receptor alpha; ROS, reactive oxygen species; THR, thyroid hormone receptor; TJPs, tight junction proteins; TNF-α, tumor necrosis factor-alpha. Image created with Biorender.com.

We propose that farnesol be considered a key mediator of communication between the gut and the brain. Farnesol, a member of the evolutionarily conserved MVA pathway, exhibits broad biological activities ranging from quorum sensing in yeast and biofilm inhibition in bacteria to neuroprotection in autoimmune and neuroinflammatory conditions. The molecular targets of farnesol include the mammalian nuclear receptor FXR and voltage-gated calcium channels, both of which are intimately involved in immune homeostasis. This makes farnesol a plausible metabolic mediator of crosstalk among brain cells, immune cells, intestinal barrier cells, and intestinal microbes. Farnesol and farnesol derivatives have received significant attention for their therapeutic potential (Gupta et al., 2019). Figure 2 illustrates our understanding of the role of farnesol and the farnesol pathway in intercommunication among gut microbes, the innate and adaptive immune systems, and the brain. However, further research and a multidisciplinary approach will be necessary to fully evaluate the therapeutic implications of the knowledge acquired thus far, particularly for the treatment of neuroinflammation and neurodegeneration.
